# The ZZ domain of HERC2 is a receptor of arginylated substrates

**DOI:** 10.1038/s41598-022-10119-w

**Published:** 2022-04-11

**Authors:** Adam H. Tencer, Jiuyang Liu, Jing Zhu, Nathaniel T. Burkholder, Yi Zhang, Wenwen Wu, Brian D. Strahl, Tomohiko Ohta, Tatiana G. Kutateladze

**Affiliations:** 1 Department of Pharmacology, University of Colorado School of Medicine, Aurora, CO 80045 USA; 2 Department of Translational Oncology, St. Marianna University Graduate School of Medicine, 2‑16‑1, Sugao, Miyamae‑ku, Kawasaki, 216‑8511 Japan; 3 Department of Biochemistry and Biophysics, The University of North Carolina School of Medicine, Chapel Hill, NC 27599 USA

**Keywords:** Proteins, Structural biology

## Abstract

The E3 ubiquitin ligase HERC2 has been linked to neurological diseases and cancer, however it remains a poorly characterized human protein. Here, we show that the ZZ domain of HERC2 (HERC2_ZZ_) recognizes a mimetic of the Nt-R cargo degradation signal. NMR titration experiments and mutagenesis results reveal that the Nt-R mimetic peptide occupies a well-defined binding site of HERC2_ZZ_ comprising of the negatively charged aspartic acids. We report the crystal structure of the DOC domain of HERC2 (HERC2_DOC_) that is adjacent to HERC2_ZZ_ and show that a conformational rearrangement in the protein may occur when the two domains are linked. Immunofluorescence microscopy data suggest that the stimulation of autophagy promotes targeting of HERC2 to the proteasome. Our findings suggest a role of cytosolic HERC2 in the ubiquitin-dependent degradation pathways.

## Introduction

Ubiquitination mediates proteasomal protein degradation, autophagic recycling and intracellular signaling. It also represents one of the major posttranslational modifications of proteins. The ubiquitination reaction is a three-step process that requires the catalytic activity of three enzymes, including the ubiquitin (Ub)-activating enzyme, E1, the Ub-conjugating enzyme, E2, and the Ub-ligase, E3. The selectivity of ubiquitination relies on E3, which recognizes a specific degradation signal in the substrate and accelerates the transfer of Ub from E2 to a lysine residue of this substrate. One of the degradation signals, the amino-terminal arginine residue (Nt-R) of the substrate, is produced by proteolytic cleavage of the protein sequence before the arginine or enzymatically added to the sequence by Arg-tRNA transferases^1–4^. Aspartic and glutamic acids are particularly susceptible to arginylation, whereas cysteine must be oxidized, and asparagine and glutamine must undergo deamidation before they can be arginylated^5^. In the Ub-mediated selective proteasomal degradation pathway, the Nt-arginylated substrates are recognized by a zinc finger motif, also known as the UBR box, of the E3 ligases UBRs (Ub ligase *N*-recognins)^4,6^. This leads to polyubiquitination of the substrates and transport them to the proteasome for degradation.

Recent biochemical and structural studies have identified another protein domain capable of recognizing the Nt-R signal. The ZZ-type zinc finger of p62 (p62_ZZ_) was shown to interact with the N-terminally arginylated substrates. p62 is a key component of autophagy, an intracellular catabolic process by which cytoplasmic components of the cell are carried to the lysosome for degradation and recycling^7^. p62 functions as a cargo-specific autophagy receptor because it associates with ubiquitinated aggregates through the UBA and ZZ domains and helps sequestering the cargo in the autophagosome vesicle. Autophagosome subsequently fuses with the lysosome where the sequestered cargo is degraded by lysosomal enzymes. Binding of the p62_ZZ_ domain to the Nt-R cargo degradation signal is necessary for p62 autophagosome targeting^8–11^.

Like the ubiquitin recognizing protein p62, the E3 ubiquitin protein ligase HERC2 contains the ZZ domain and shuttles between the nucleus and the cytoplasm^12–14^. The nuclear pool of HERC2 has been shown to play a role in DNA replication, checkpoint control and DNA damage repair processes. In response to DNA damage, HERC2 undergoes SUMOylation and interacts with RNF8, another E3 ubiquitin ligase, which ubiquitinates histone substrates, recruiting repair factors to DNA damage foci and promoting DNA repair^15^. HERC2 was shown to be necessary for the nucleolar localization and functions of the BML and WRN helicases^16,17^. However, a limited information is available regarding the function of the cytosolic pool of HERC2. HERC2 is implicated in endosomal trafficking^18^, modulation of centrosome architecture^19^, and mediating polyubiquitination and proteasomal degradation of proteins, such as USP33^20^ and FBXL5, an essential component of mammalian iron homeostasis^21^. HERC2 also interacts with, ubiquitinates, and regulates the cell cycle checkpoint activity and stability of the breast cancer suppressor BRCA1^13^.

**Figure 1:**
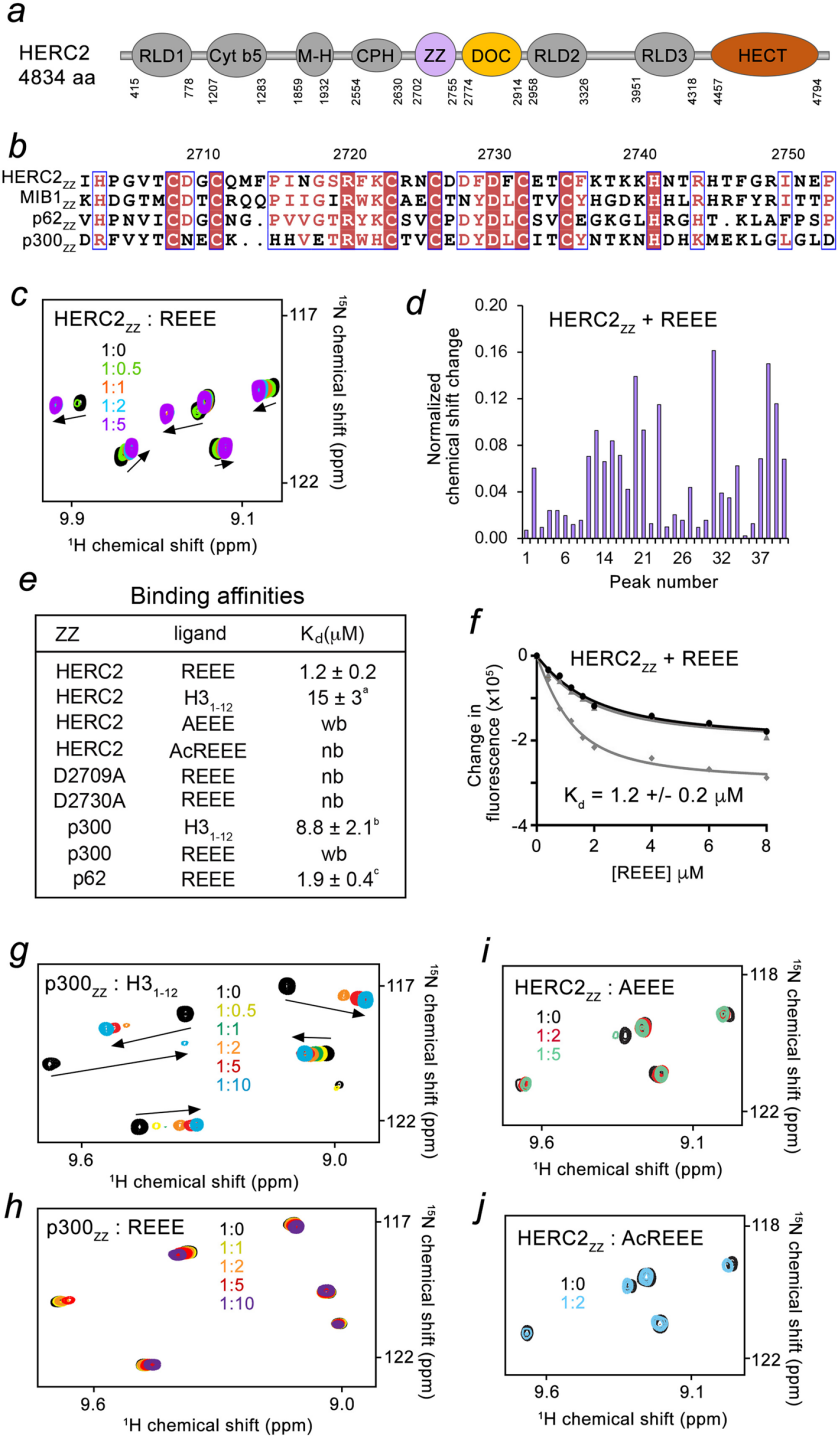
HERC2_ZZ_ binds to the Nt-R degradation signal. (**a**) HERC2 domain architecture. The presence of the cytochrome b5-like motif (Cyt b5), the mind-bomb/HERC2 (M-H) domain, the CPH domain, the ZZ domain and the DOC domain distinguishes HERC2 from other members of the HERC family of proteins (HERC1 and HERC3-6). (**b**) Alignment of the amino acid sequences of the ZZ domains from HERC2, MIB1, p62 and p300. (**c**) Superimposed ^1^H,^15^N HSQC spectra of ^15^N-labeled HERC2_ZZ_ collected while the REEE peptide was titrated in the NMR sample. Spectra are color coded according to the protein:peptide molar ratio. (**d**) A plot of normalized chemical shift change (peaks are unassigned) induced in HERC2_ZZ_ by the REEE peptide at a 1:5 protein:peptide molar ratio. Peak numbering is shown in Supplementary Table 1. (**e**) Binding affinities for the indicated ZZ domains and ligands. (^a^), (^b^) and (^c^) values are taken from Refs.^8,24,32^. *Wb* weak binding, *nb* no binding. (**f**) Binding curves used to determine K_d_ for the interaction of HERC2_ZZ_ with the REEE peptide by tryptophan fluorescence. The K_d_ value was averaged over three separate experiments, with error calculated as the standard deviation between the runs. (**g**,**h**) Overlay of ^1^H,^15^N HSQC spectra of p300_ZZ_ collected before (black) and after the addition of the H3_1–12_ peptide (**g**), or REEE peptide (**h**). Spectra are color coded according to the protein:peptide molar ratio. (**i**,**j**) Overlay of ^1^H,^15^N HSQC spectra of HERC2_ZZ_ collected before (black) and after the addition of the AEEE peptide (**i**) or AcREEE peptide (**j**). Spectra are color coded according to the protein:peptide molar ratio.

The *HERC2* gene encodes a large, 4834 residue protein that in addition to the ZZ domain contains three RCC1-like domains (RLDs), a cytochrome b5-like motif (Cyt b5), a mind-bomb/HERC2 (M-H) domain, a CPH domain, a DOC domain (HERC2_DOC_), and the carboxy-terminal catalytic E3 ubiquitin ligase HECT domain (Fig. 1a). Although *HERC2* was identified in 1998^22^, progress in defining biological functions of the HERC2 domains remains slow, likely, due to its gigantic size. Our recent work shows that the ZZ domain of nuclear HERC2 binds to the amino-terminal sequences of histone H3 and SUMO1^23,24^. Here, we demonstrate that the ZZ domain of HERC2 (HERC2_ZZ_) recognizes the Nt-R degradation signal, which suggests a role of cytosolic HERC2 in the selective cargo degradation pathways. We employ NMR experiments to explore the relationship between the neighboring ZZ and DOC domains in HERC2 and report the crystal structure of HERC2_DOC_.

## Results and discussion

### HERC2_ZZ_ recognizes the Nt-R degradation signal

The ZZ domains of nine human proteins have been identified as readers of the amino-terminus of histone H3 tail^25^. Some of these proteins, including p62, HERC2 and p300/CBP, are found in both nuclear and cytosolic fractions, and some, like KCMF1 and MIB1/2, localize primarily to the cytoplasm of the cell. We showed that in the cytoplasm, the ZZ domain of p62 (p62_ZZ_) binds to the Nt-R cargo degradation signal, and this interaction is essential in the autophagic function of p62^8^. A high conservation of the amino acid sequences of the ZZ domains from ubiquitin-recognizing p62 and the ubiquitin ligase HERC2 suggested a similar biological activity for p62_ZZ_ and HERC2_ZZ_ (Fig. 1b). To explore whether the recognition of the Nt-R degradation signal is conserved in HERC2, we produced ^15^N-labelled HERC2_ZZ_ and monitored its interaction with the mimetic of Nt-R, the REEE peptide, by NMR spectroscopy. ^1^H,^15^N HSQC (heteronuclear single quantum coherence) spectra of HERC2_ZZ_ were recorded while the REEE peptide was added stepwise to the NMR sample. Substantial chemical shift perturbations (CSPs) in the spectra of HERC2_ZZ_, induced by the peptide, indicated formation of the complex (Fig. 1c,d and Supplementary Table 1). A number of amide crosspeaks of the HERC2_ZZ_ apo-state disappeared upon addition of the peptide, and another set of resonances corresponding to the bound state appeared. The slow-to-intermediate exchange regime on the NMR timescale suggested tight binding (Fig. 1c and Supplementary Fig. 1), which was confirmed through measuring the dissociation constant (K_d_) for the interaction of HERC2_ZZ_ with the REEE peptide by tryptophan fluorescence (K_d_ = 1.2 μM) (Fig. 1e,f).

We note that in contrast to HERC2_ZZ_ and p62_ZZ_, which have binding partners in both nucleus and cytoplasm of the cell, the ZZ domain of p300 (p300_ZZ_) shows only nuclear activity and recognizes the histone H3_1–12_ (aa 1–12 of H3) peptide (Fig. 1e,g). Titration of the REEE peptide caused very small CSPs in the ^1^H,^15^N HSQC spectrum of p300_ZZ_, implying that cytosolic p300 does not act as a degradation pathway receptor (Fig. 1h). Together, NMR experiments demonstrate that the ZZ domain of HERC2 (but not of p300) recognizes the Nt-R cargo degradation signal, and thus the cytosolic pool of HERC2 could be involved in selective substrate degradation and/or recycling processes.

### The Nt-R mimetic occupies the acidic site of HERC2_ZZ_

**Figure 2:**
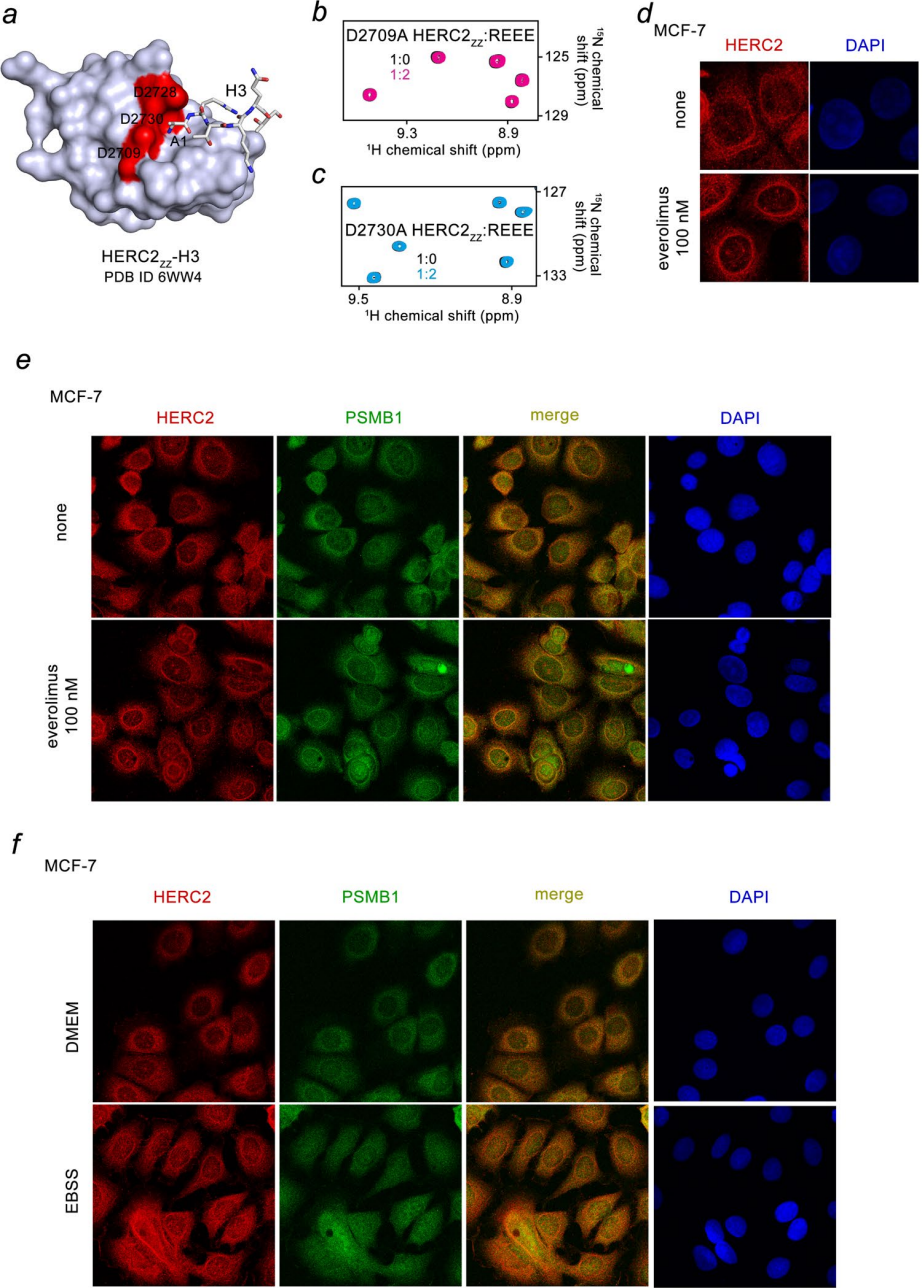
HERC2 is involved in the degradation pathways. (**a**) The surface representation of the crystal structure of HERC2_ZZ_ in complex with the H3 peptide (PDB: 6WW4). The H3 peptide is shown as grey sticks, and the negatively charged binding site residues of HERC2_ZZ_ are colored red. (**b**,**c**) Superimposed ^1^H,^15^N HSQC spectra of the mutated HERC2_ZZ_ collected while the REEE peptide was titrated in the NMR samples. Spectra are color coded according to the protein:peptide molar ratio. (**d**–**f**) MCF-7 cells incubated with or without 100 nM everolimus for 24 h (**d**,**e**), or either the DMEM or EBSS medium for 4 h (**f**) were subjected to immunostaining with the indicated antibodies. The nuclei were counter stained with DAPI.

To determine the role of the first arginine residue of the Nt-R signal in the formation of the HERC2_ZZ_-Nt-R complex, we tested the peptide in which Arg1 was replaced with an alanine. Small CSPs in HERC2_ZZ_, observed upon addition of a fivefold excess of the AEEE peptide, indicated that the binding was substantially reduced (Fig. 1i). Furthermore, the binding was essentially abolished when the α-amino terminal NH_3_^+^ group of Arg1 was blocked by acetylation in the Ac-REEE peptide (Fig. 1j). It has been shown that the N-terminus of the histone H3 sequence (ARTK) is bound in the negatively charged pocket of HERC2_ZZ_, comprising D2709, D2728 and D2730 (Fig. 2a, red)^24^. Mutation of D2709 or D2730 to an alanine eliminated binding of HERC2_ZZ_ to either the histone H3_1-12_ peptide^24^ or the REEE peptide (Figs. 1e, 2b,c). Furthermore, a similar set of crosspeaks in ^1^H,^15^N HSQC spectrum of HERC_ZZ_ was perturbed by REEE peptide or H3_1–12_ peptide (Supplementary Fig. 1 and Ref.^24^). Collectively, these data point to a critical role of the free, unprotected Arg1 residue in recognition of the Nt-R signal by HERC2_ZZ_ and also reveal that both nuclear and cytoplasmic ligands of HERC2_ZZ_ occupy the same acidic binding pocket of the protein.

### HERC2 is involved primarily in the proteasomal degradation pathway

To understand the role of the cytosolic HERC2 E3 Ub ligase in the degradation pathways we stimulated autophagy via treating MCF-7 and HeLa cells with the mTOR inhibitor everolimus or starving the cells and visualized endogenous HERC2 by immunofluorescence. Although HERC2 could be engaged with an autophagosome through the interaction with NCOA4, a selective cargo receptor for the autophagic turnover of iron located on the surface of autophagosomes^26^, a weak co-localization of HERC2 with LC3, an autophagic marker, indicated that it could be only a minor function of HERC2 (Supplementary Fig. 2a). Treatment of MCF-7 cells with everolimus led to a notable change in localization of HERC2 and its accumulation around the nucleus (Fig. 2d), whereas immunostaining using antibodies against the 20S proteasome β1 subunit (PSMB1) showed a high degree co-localization of HERC2 with this mark of the proteasomal degradation pathway (Fig. 2e). The change in HERC2 localization around the nuclear surface upon stimulation of autophagy by everolimus, coincided with the change in PSMB1 localization. Likewise, Earle’s balanced salts solution (EBSS)-induced starvation caused similar changes in localization of HERC2 and PSMB1 in both MCF-7 and HeLa cells (Fig. 2f and Supplementary Fig. 2b). These findings suggest that the stimulation of autophagy promotes targeting of HERC2 to the proteasome.

### The HERC2_ZZ_ activity is unaffected by HERC2_DOC_

**Figure 3:**
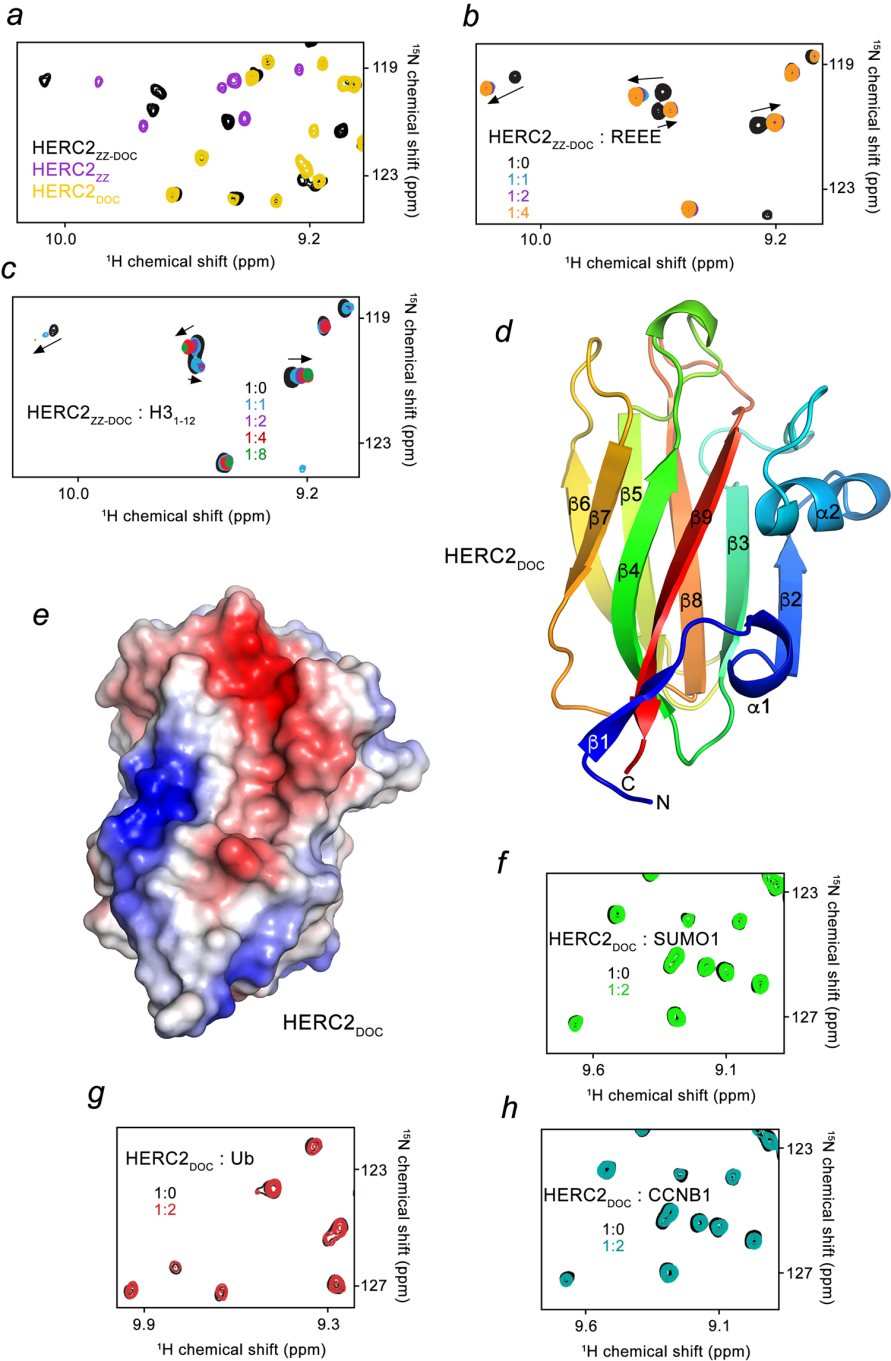
HERC2_ZZ_ function is unaffected by HERC2_DOC_. (**a**) Superimposed ^1^H,^15^N HSQC spectra of HERC2_ZZ_ (purple), HERC2_DOC_ (yellow), and HERC2_ZZ-DOC_ (black). (**b**,**c**) Superimposed ^1^H,^15^N HSQC spectra of HERC2_ZZ-DOC_ collected while the REEE peptide (**b**) or the H3_1–12_ peptide (**c**) was titrated in the NMR sample. Spectra are color coded according to the protein:ligand molar ratio. (**d**) A ribbon diagram of the crystal structure of apo HERC2_DOC_, shown in rainbow shades from blue (the N-terminus) to red (the C-terminus). (**e**) Electrostatic surface potential of HERC2_DOC_ is colored blue and red for positive and negative charges, respectively. (**f**–**h**) Overlay of ^1^H,^15^N HSQC spectra of HERC2_DOC_ collected before (black) and after the addition of full length SUMO1 (**f**), Ub (**g**) or CCNB1 peptide (**h**). Spectra are color coded according to the protein:ligand molar ratio.

HERC2_ZZ_ is followed by HERC2_DOC_ of unknown structure and function. A substantial dispersion of amide resonances in the ^1^H,^15^N HSQC spectrum of HERC2_DOC_ indicates that this domain is folded and stable (Fig. 3a, yellow). We overlayed ^1^H,^15^N HSQC spectra of HERC2_DOC_, HERC2_ZZ_, and the construct encompassing both ZZ and DOC domains (HERC2_ZZ-DOC_) and noticed that many crosspeaks of the individual domains do not overlap with the crosspeaks of the linked construct (Supplementary Fig. 3). While this could be due to a direct interaction between the domains, the absence of CSPs in ^15^N-labeled HERC2_DOC_ upon titration with unlabeled HERC2_ZZ_ argued against this notion (Supplementary Fig. 4a). Additionally, we found that HERC2_DOC_ does not interact with the ligands of HERC2_ZZ_, as no CSPs were induced in HERC2_DOC_ by either the REEE peptide or H3_1-12_ peptide (Supplementary Fig. 4b,c) and either linked HERC2_ZZ-DOC_ or isolated HERC2_ZZ_ and HERC2_DOC_ remain monomeric in solution (Supplementary Fig. 5). Furthermore, the presence of HERC2_DOC_ in the linked HERC2_ZZ-DOC_ construct does not alter the binding of HERC2_ZZ_ to these ligands (Fig. 3b,c). The binding affinity of HERC2_ZZ_ to the REEE peptide in the presence of HERC2_DOC_ remained unchanged (K_d_ of 1 µM, Supplementary Fig. 4d). We concluded that although HERC2_DOC_ and HERC2_ZZ_ do not appreciably interact, a conformational rearrangement in the protein may occur when the two domains are linked.

### The crystal structure of HERC2_DOC_

To characterize HERC2_DOC_, we crystallized this domain and determined its crystal structure, refining it to a 2 Å resolution (Table 1). The HERC2_DOC_ structure consists of nine antiparallel β-strands and two short α-helices (Fig. 3d). The core of the domain folds into a β-sandwich with the five strands, β2, β3, β8, β5 and β6 forming one β-sheet that packs against another β-sheet containing the four strands, β1, β9, β4 and β7. The first α-helix links β1 and β2 strands and another α-helix, α2, is located between β2 and β3 strands. One of the open ends of the sandwich is delineated by the short loops connecting the β strands, whereas the opposite end is surrounded by the longer loops (Fig. 3d, bottom and top, respectively). Electrostatic surface potential of HERC2_DOC_ reveals a well-defined negatively charged groove that lays parallel to the β9 strand and is ideally positioned for pairing with an additional positively charged β-strand of a ligand (Fig. 3e). Because histone H3 regions are highly positively charged and are known to adopt the β-strand conformation in several complexes, we tested whether HERC2_DOC_ can bind any of the H3 regions using a pull-down assay. As shown in Supplementary Fig. 4e, HERC2_DOC_ does not recognize any of the H3 peptides tested, or other potential ligands, including SUMO1, Ub and the D-box region of CCNB1 (Fig. 3f–h)^24,27^.

**Table 1:** Data collection and refinement statics for the crystal structure of HERC2_DOC_.

PDB ID	HERC2 DOC domain
	7RGW
**Data collection**	
Space group	*P* 6_5_
**Cell dimensions**	
a, b, c (Å)	89.37, 89.37, 52.85
α, β, γ (°)	90.00, 90.00, 120.00
Wavelength (Å)	1.54
Resolution^a^ (Å)	29.25–1.99 (2.05–1.99)
Completeness (%)	99.8 (99.8)
Redundancy	4.7 (3.0)
*R*_sym_ or *R*_merge_ (%)	8.4 (32.5)
CC_1/2_ in highest shell	(0.795)
*I*/σ*I*	17.8 (4.1)
**Refinement**	
Resolution (Å)	29.35–1.99
Unique reflections	16,610
*R*_work_/*R*_free_	18.14/21.35
**Rooot-mean-square deviation**	
Bond lengths (Å)	0.007
Bond angles (°)	0.843l
***B*-factors (Å**^**2**^**)**	
Protein	20.8
Water	31.61
**No. atoms**	
Protein	1097
Water	183
**Ramachandran plot (%)**	
Favored/allowed/outlier	97.8/2.2/0.0

^a^Values in parentheses are for highest-resolution shell.

Little information is available about the DOC domain family, which consists of only four proteins^27–30^. The structure of the DOC domain (originally referred to as the APC10 domain) of the protein APC10^28^ superimposes with the structure of HERC2_DOC_ with an r.m.s.d. of 0.9 Å (Supplementary Fig. 6). We note that despite the DOC domain structure was reported 20 years ago^28^, a ligand of this domain remains unknown. Since similarly to HERC2, APC10 is an E3 ubiquitin ligase, the DOC domain might be involved in the ubiquitination-dependent targeted degradation or recycling processes.

## Concluding remarks

Our data demonstrate that the ZZ domain of HERC2 plays critical roles in function of both nuclear and cytoplasmic pools of HERC2. In the nucleus, HERC2_ZZ_ binds to the amino-terminal sequences of histone H3 and SUMO1^23,24^, which is essential in mediating conformational changes, DNA binding activity, chromatin localization and catalytic function of HERC2. In the cytoplasm, HERC2_ZZ_ recognizes the Nt-R degradation signal, and this interaction suggests that cytosolic HERC2 could act as a selective cargo degradation or recycling receptor. The ability to associate with specific binding partners in the nucleus and cytoplasm is conserved in p62_ZZ_ and HERC2_ZZ_ but not in p300_ZZ_, which does not bind the Nt-R signal. The ZZ domain of the yeast protein Nbr1, a homolog of a human selective autophagy receptor, has been shown to bind the N-termini of the specific cargo proteins Ams1 and Ape4 and is also engaged with other regions of these proteins^31^. The atomic-resolution structures of the ZZ domains of HERC2, p62, p300, ZZZ3 and Nbr1 (Nbr1_ZZ_) in complex with their ligands and biochemical analyses reveal a common mechanism for the ligand recognition^8–10,25,32–34^. The acidic binding site of the ZZ domains (Supplementary Fig. 7) accommodates the positively charged amino-terminal group of the first residue in all ligands. Notably, even though the sequences of the binding partners are diverse—HERC2_ZZ_ binds the ART, SDQ and REE sequences of H3, SUMO1 and the Nt-R signal, respectively, whereas Nbr1_ZZ_ binds the TL and MQL sequences of Ams1 and Ape4, respectively—these interactions are cargo specific: mutations of the residues in the ligands reduce or eliminate these interactions. It will be interesting in future studies to determine the selectivity of other cytosolic ZZ domain-containing proteins. It will also be important to identify the function of the DOC domain family, including HERC2_DOC_ and explore the importance of HERC2_ZZ-DOC_ in the catalytic activity of HERC2.

## Experimental procedures

All methods were carried out in accordance with relevant guidelines and regulations.

### Protein expression and purification

Human HERC2_ZZ_ (aa 2702–2755) and HERC2_ZZ-DOC_ (aa 2702–2914) were cloned into a pCIOX vector with the N-terminal His_8x_-SUMO tag and the Ulp1 cleavage site. In order to quantify the protein and perform tryptophan fluorescence assay, an additional tryptophan residue was introduced at the C-terminus of HERC2_ZZ_. HERC2_DOC_ (aa 2759–2914) was cloned into a pET28MHL vector with the His_6x_-tag and the TEV cleavage site. The human SUMO1 protein was cloned into a pDEST-15 vector with the N-terminal GST tag and the TEV cleavage site. Proteins were expressed in *E. coli* BL21 (DE3) RIL cells grown in either LB or M9 minimal media supplemented with ^15^NH_4_Cl (Sigma-Algrich) and 0.05 mM ZnCl_2_ (for HERC2_ZZ_ and HERC2_ZZ-DOC_). Following induction with 0.5 mM IPTG for 20 h at 16 °C, cells were harvested by centrifugation and lysed by sonication. The His_6x_-tag and His_8x_-SUMO tagged proteins were purified on HisPur Ni–NTA resin (Thermo) in 50 mM Tris–HCl (pH 7.5) buffer, supplemented with 500 mM NaCl, and 5 mM β-mercaptoethanol. The His_6x_-tagged HERC2_DOC_ was eluted with increasing gradient of imidazole. The His_8x_-SUMO tagged protein was cleaved overnight at 4 °C with the ULP1 protease. Unlabeled proteins were further purified by size exclusion chromatography and concentrated in Millipore concentrators. All mutants were generated by site-directed mutagenesis using the Stratagene QuikChange mutagenesis protocol, then grown and purified as wild-type proteins.

### NMR experiments

NMR experiments were carried out at 298 K on a Varian INOVA 600 spectrometer as described^35^. NMR samples contained 0.1–0.2 mM uniformly ^15^N-labled WT and mutant HERC2_ZZ_, HERC2_ZZ-DOC_ or HERC2_DOC_ in either 20 mM Tris (pH 6.8) or 20 mM PBS (pH 6.5) buffer supplemented with 150 mM NaCl, 2 mM DTT and 10% D_2_O. Binding was characterized by monitoring chemical shift changes in the ^15^N-labeled proteins induced by the unlabeled proteins or REEE, AEEE and AcREEE peptides (synthesized by SynPeptide). Ub was purchased from R&D systems, Inc. Normalized chemical shift change in Fig. 1d was calculated as $\Delta \delta =\sqrt{{\left(\Delta \delta H\right)}^{2}+{\left(\Delta \delta N/5\right)}^{2}} ,$ where δ is the chemical shift in parts per million (ppm).

### X-ray crystallography

Purified HERC2_DOC_ (aa 2759–2914) was concentrated to 6.5 mg/mL. HERC2_DOC_ crystals were obtained at 18 °C using the sitting drop vapor diffusion method. 1 μL protein solution was mixed with 1 μL reservoir that contained 0.1 M HEPES, pH 7.5 and 25% PEG3350. Crystals were cryoprotected with the addition of 25% ethylene glycol before being flash-frozen in liquid nitrogen. X-ray diffraction data were collected on a Rigaku Micromax 007 high-frequency microfocus X-ray generator at the CU Anschutz X-ray crystallography core facility. HKL2000 was used for indexing, scaling and data reduction^36^. The structure was determined by the Phaser-MR program in Phenix using APC10 (PDB code: 1JHJ) as a search model. Model building was performed with Coot^37^, and the structure was refined with Phenix Refine^38^. Residues 2774–2914 of HERC2 were modeled to the electron density map, whereas the electron density for the His_6_ tag with a TEV cleavage site and residues 2759–2773 of HERC2 were not observed likely because of flexibility. The crystallographic and refinement statistics are summarized in Table 1.

### Tryptophan fluorescence

Spectra were recorded at 25 °C on a Fluoromax Plus-C spectrofluorometer (HORIBA). The samples containing 1 μM HERC2_ZZ_ (aa 2702–2755 with additional tryptophan at the C-terminus) (with or without 1 μM HERC2_DOC_) in 20 mM Tris (pH 6.8), 150 mM NaCl, 2 mM DTT and progressively increasing concentration of the REEE peptide were excited at 295 nm. Emission spectra were recorded between 320 and 360 nm with a 0.5 nm step size and a 0.5 s integration time and averaged over three scans. The K_d_ values were determined using a nonlinear least-squares analysis and the equation:

$$\Delta I={\mathrm{\Delta I}}_{max}\frac{\left(\left(\left[L\right]+\left[P\right]+{K}_{d}\right)- \sqrt{{\left(\left[L\right]+\left[P\right]+{K}_{d}\right)}^{2}-4[P][L])}\right)}{2[P]},$$

where [L] is the concentration of the peptide, [P] is the concentration of the protein, ΔI is the observed change of signal intensity, and ΔI_max_ is the difference in signal intensity of the free and bound states of the protein. The K_d_ values were averaged over three separate experiments with error calculated as the standard deviation between the runs.

### Immunofluorescence microscopy

HeLa and MCF-7 cells were obtained from ATCC with authentication and stored in liquid nitrogen or cultured according to the supplier’s instructions for less than 20 passages. For indirect immunofluorescence labeling of cells and fluorescence detection, cells were fixed and permeabilized with cold methanol and acetone, respectively, as described previously^39^. Cells were then washed, blocked with 3% goat serum and 0.1% Triton X-100, and labeled with primary and fluorescent-labeled secondary antibodies. The slides were mounted with the ProLong Gold Antifade Mountant with DAPI (Invitrogen) and examined with a confocal laser-scanning microscope (LSM 510, Carl Zeiss, Germany). Rabbit polyclonal antibody against HERC2, mouse monoclonal antibodies against LC3 and PSMB1 were purchased from Bethyl Laboratories (A301-905A), MBL (4E12), and Santa Cruz Biotechnology (D-9), respectively.

### In-solution peptide pull-down assays

A total of 50 pmol of GST-tagged HERC2_DOC_ was incubated with 500 pmol of biotinylated histone peptides overnight at 4 °C rotating in peptide binding buffer (50 mM Tris pH 8.0, 150 mM NaCl, 0.1% Nonidet P-40). Following incubation, 5 μL of packed streptavidin-coated magnetic beads (Pierce) per reaction were pre-equilibrated in peptide binding buffer and then incubated with the protein-peptide mixture for 1 h at 4 °C rotating. The beads were washed 3× with peptide binding buffer using a magnetic rack followed by 5 min rotations at 4 °C, and bound complexes were eluted in 50 μL of 1× Laemmli SDS loading buffer. Samples including a 2% pulldown input, a beads + protein only negative control, and input-equivalent volumes of peptide pulldown eluates were resolved on an 8% SDS polyacrylamide gel and semi-dry transferred to a PVDF membrane. The membrane was blocked in 1× TBST with 5% non-fat dry milk and probed with anti-GST (EpiCypher, 13-0022) at 1:5000 in blocking buffer at 4 °C overnight with rotation. The blot was washed 3 × 5 min each with 1X TBST followed by incubation with anti-Rabbit-HRP (GE, NA934V) at 1:20,000 in 1× TBST for 1 h at room temperature. The membrane was then washed 3 × 5 min each with 1× TBST followed by incubation with chemiluminescent substrate as per the manufacturer’s protocol (GE, RPN2232) and detection on a ChemiDoc MP (Biorad).

## Supplementary Information

Supplementary Table 1.

Supplementary Figures.

## Data Availability

Coordinates and structure factors have been deposited in the Protein Data Bank under the accession number 7RGW. All other relevant data supporting the key findings of this study are available within the article and its Supplementary Information files or from the corresponding authors upon reasonable request.
