# Supplementary material for: The ZZ domain of HERC2 is a receptor of arginylated substrates
Source: Sci Rep. 2022 Apr 11;12:6063. doi: 10.1038/s41598-022-10119-w (PMC9001736; doi:10.1038/s41598-022-10119-w)
Supplement: Supplementary file 2 — Supplementary Figures. [file 41598_2022_10119_MOESM2_ESM.pdf]

Supplementary Information

The ZZ domain of HERC2 is a receptor of arginylated substrates

Adam H. Tencer[1,4], Jiuyang Liu[1,4], Jing Zhu[2], Nathaniel T. Burkholder[3], Yi Zhang[1], Wenwen Wu[2], Brian D. Strahl[3], Tomohiko Ohta[2] and Tatiana G. Kutateladze[1,*]

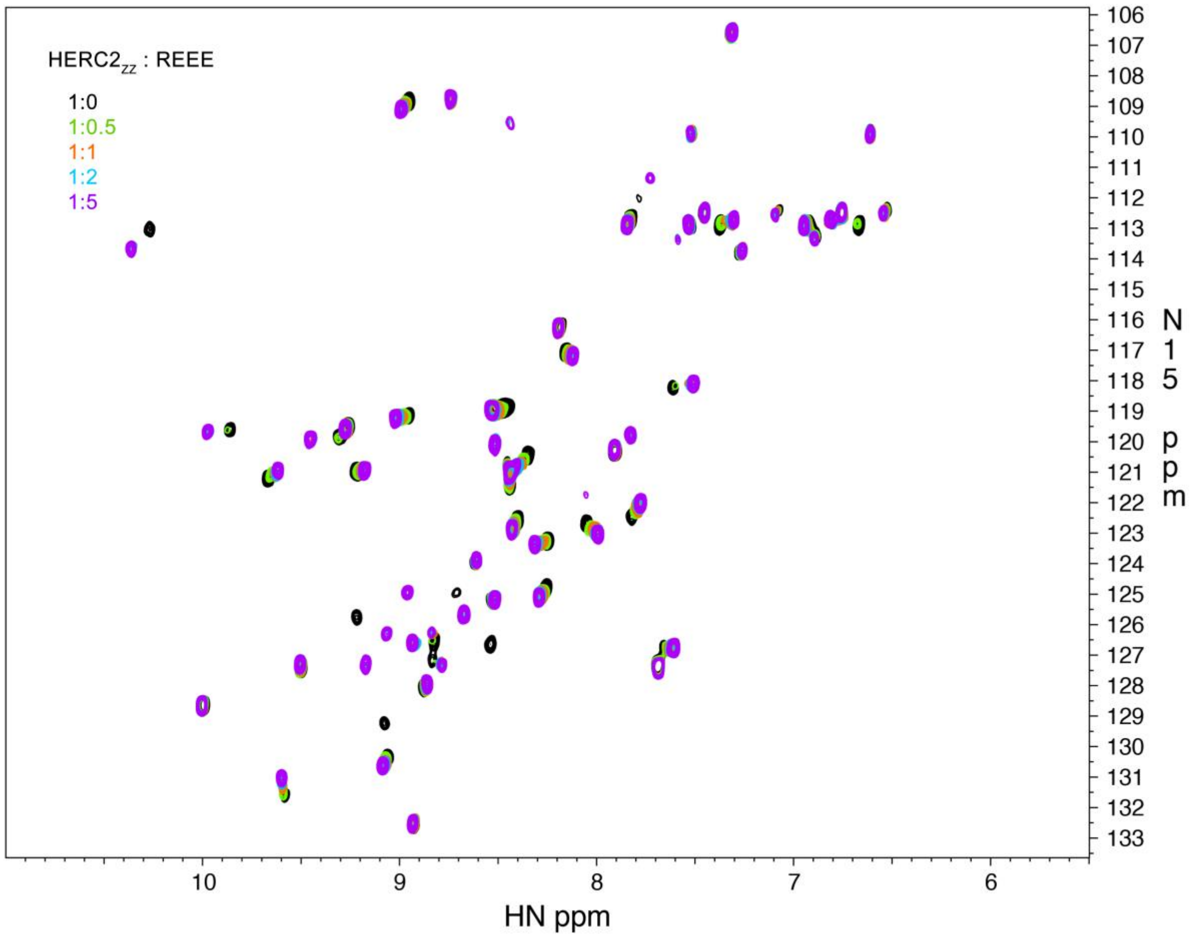

Supplementary Figure 1. Superimposed ^{1}H, ^{15}N HSQC spectra of $HERC2_{ZZ}$ collected while the REEE peptide was titrated in the NMR sample. Spectra are color coded according to the protein:peptide molar ratio.

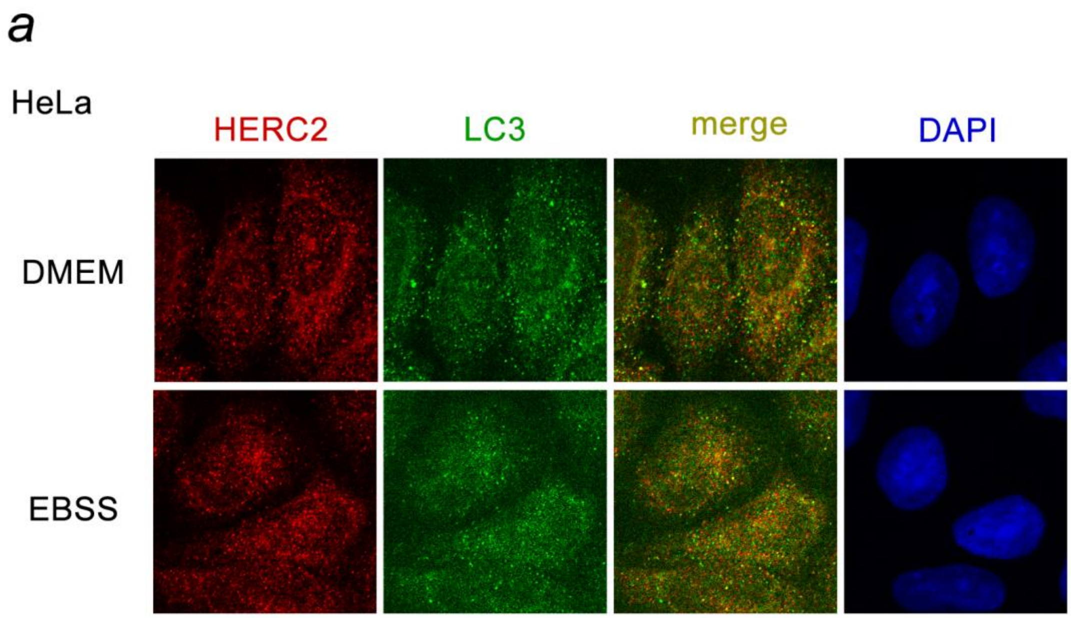

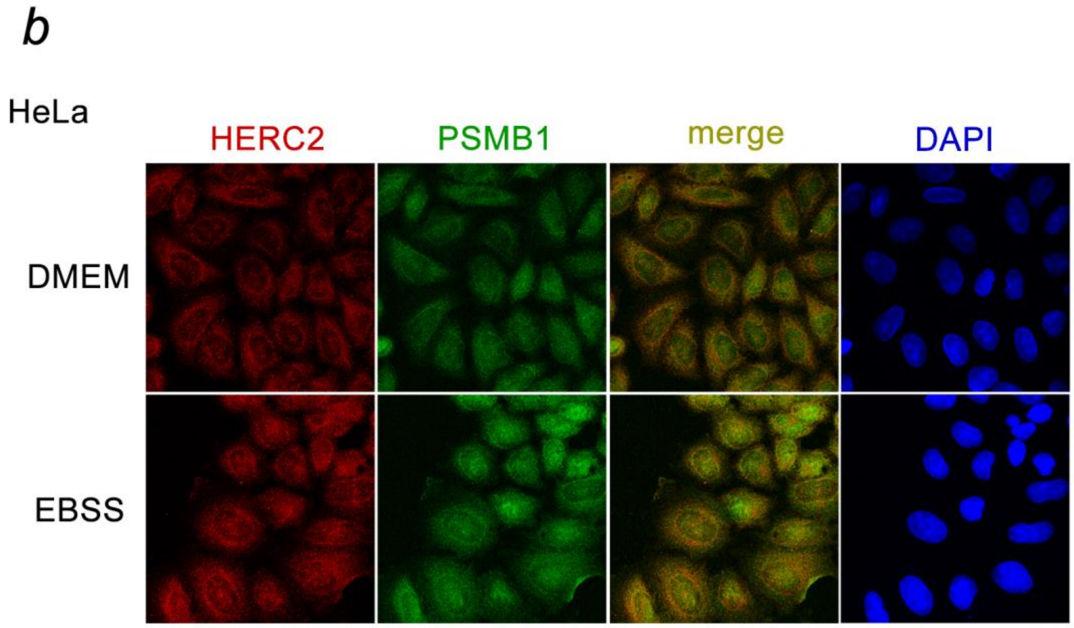

Supplementary Figure 2. HeLa cells incubated in DMEM or EBSS for 4 hours were subjected to immunostaining with antibodies against HERC2, LC3 (a) and PSMB1 (b). The nuclei were counter stained with DAPI.

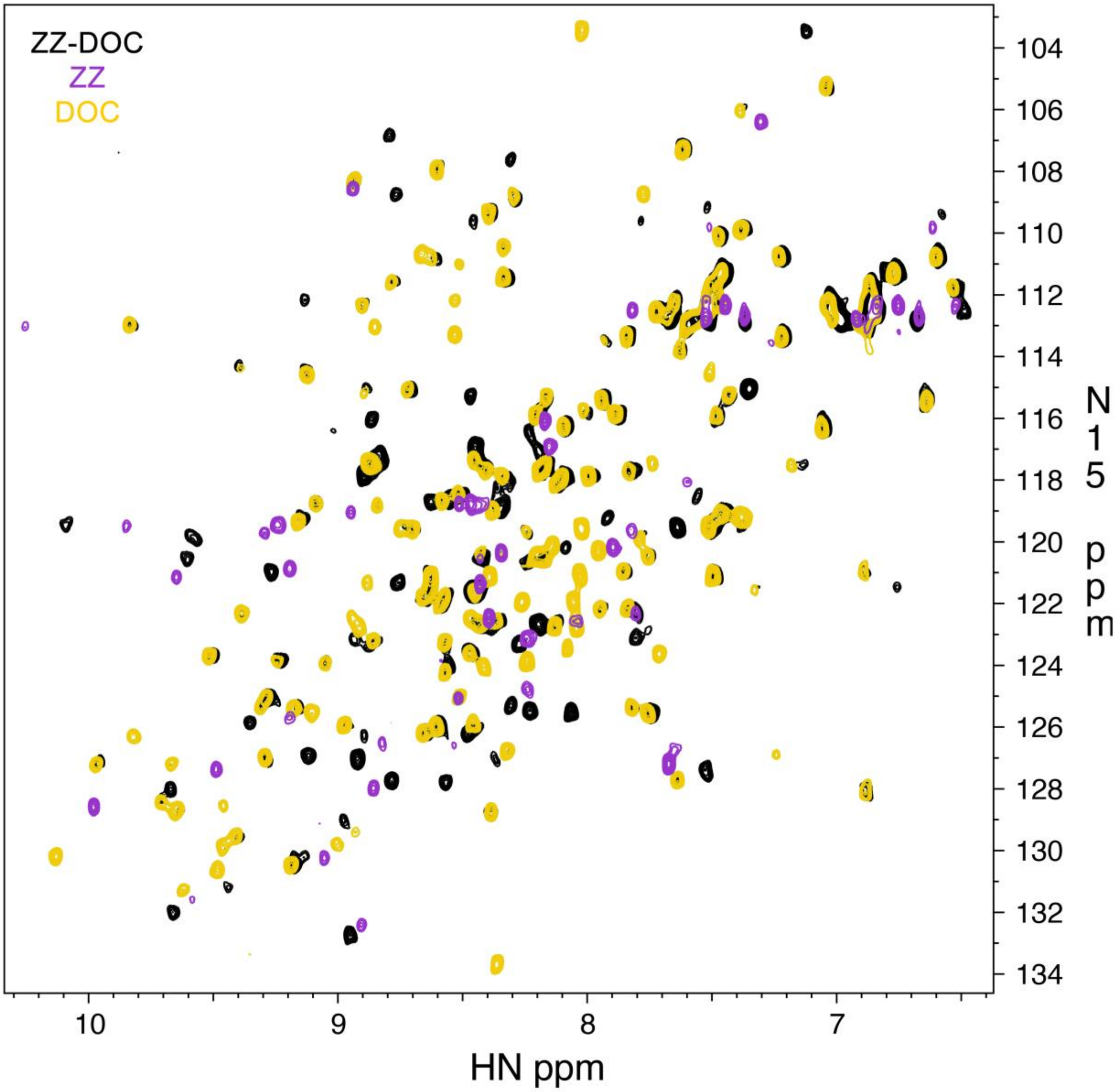

Supplementary Figure 3. (a) Superimposed ^{1}H, ^{15}N HSQC spectra of $HERC2_{ZZ}$ (purple), $HERC2_{DOC}$ (yellow), and $HERC2_{ZZ\text{-}DOC}$ (black).

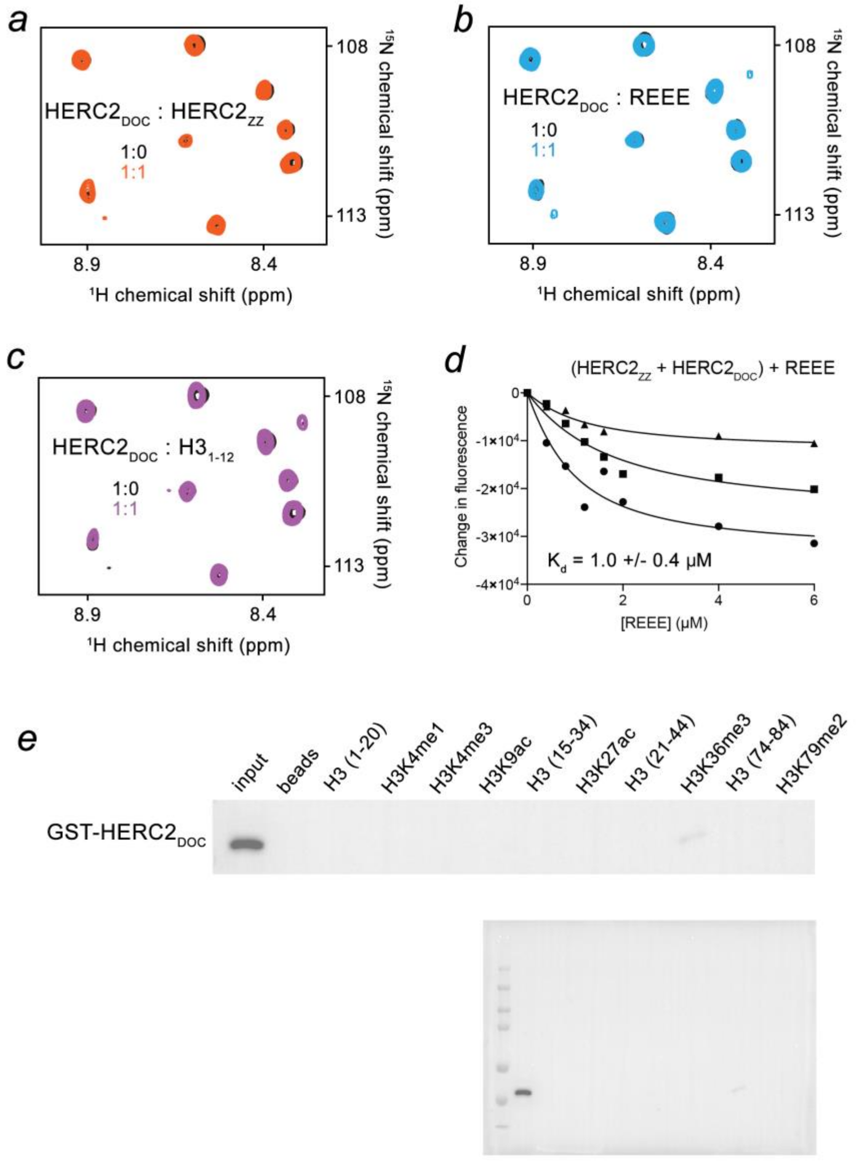

Supplementary Figure 4. (a-c) Superimposed ^{1}H,^{15}N HSQC spectra of $HERC2_{DOC}$ collected while $HERC2_{ZZ}$ (a), the REEE peptide (b) or the $H3_{1-12}$ peptide (c) was titrated in the NMR sample. Spectra are color coded according to the protein:ligand molar ratio. (d) Binding curves used to determine K_d for the interaction of $HERC2_{ZZ}$ with the REEE peptide in the presence of $HERC2_{DOC}$ (1:1 ratio of $HERC2_{ZZ}$: $HERC2_{DOC}$) by tryptophan fluorescence. The K_d value was averaged over three separate experiments, with error calculated as the standard deviation between the runs. (e) Peptide pull-down assays of GST-$HERC2_{DOC}$ with indicated histone peptides, biotinylated at their C-termini. Uncropped gel is shown below.

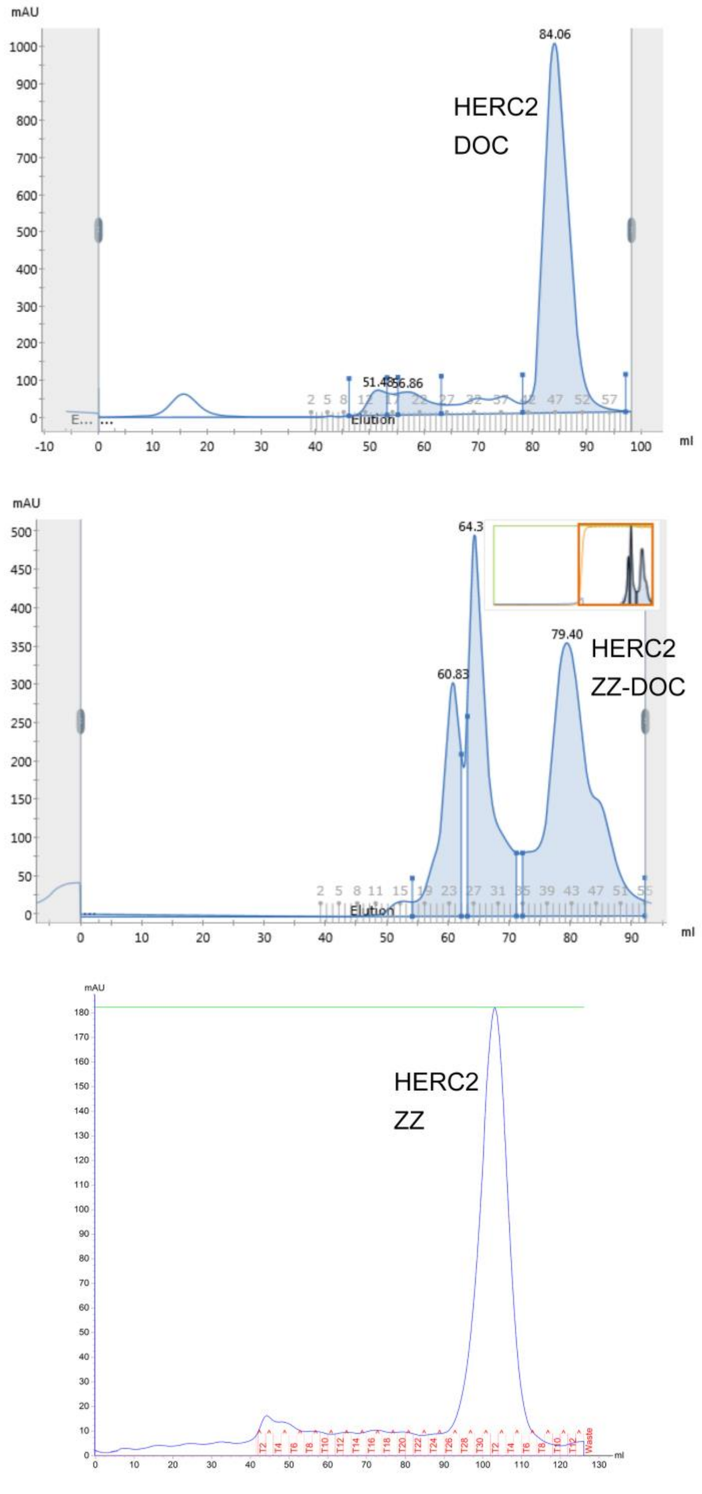

Supplementary Figure 5. Size exclusion FPLC chromatograms with elution peaks for 18 kDa HERC2$_{DOC}$ (top), 24 kDa HERC2$_{ZZ\text{-}DOC}$ (middle) and 6 kDa HERC2$_{ZZ}$ (bottom) labeled.

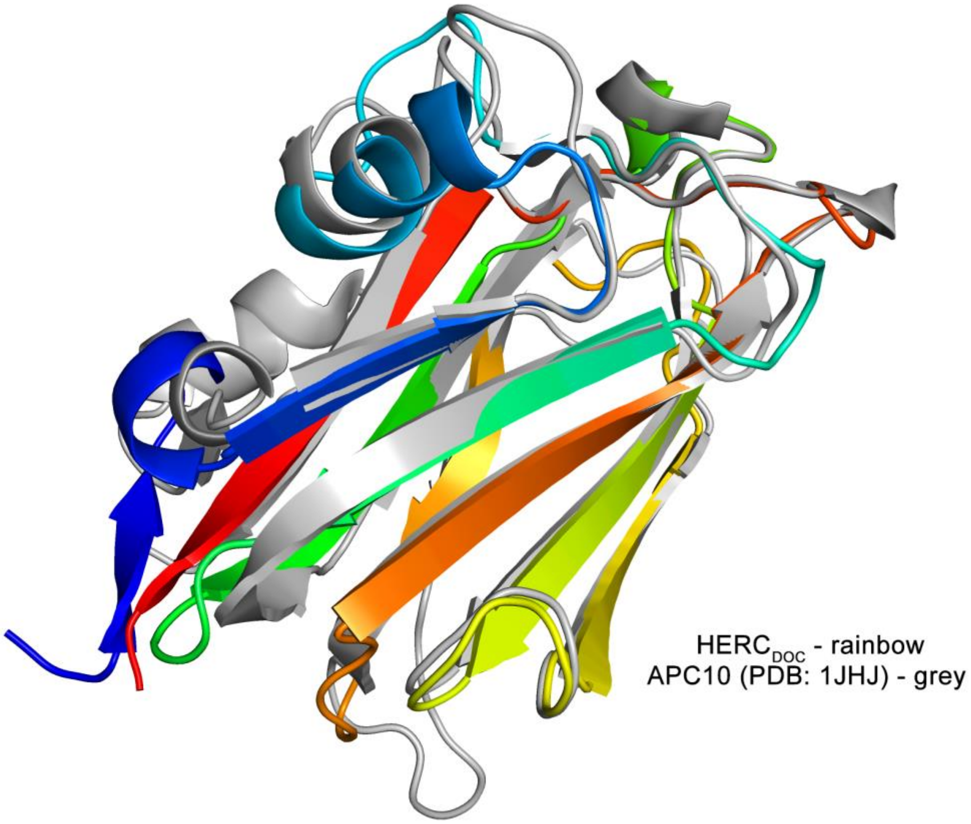

Supplementary Figure 6. Structural overlay of HERC2$_{DOC}$ (colored rainbow) and APC10 (PDB 1JHJ, grey).

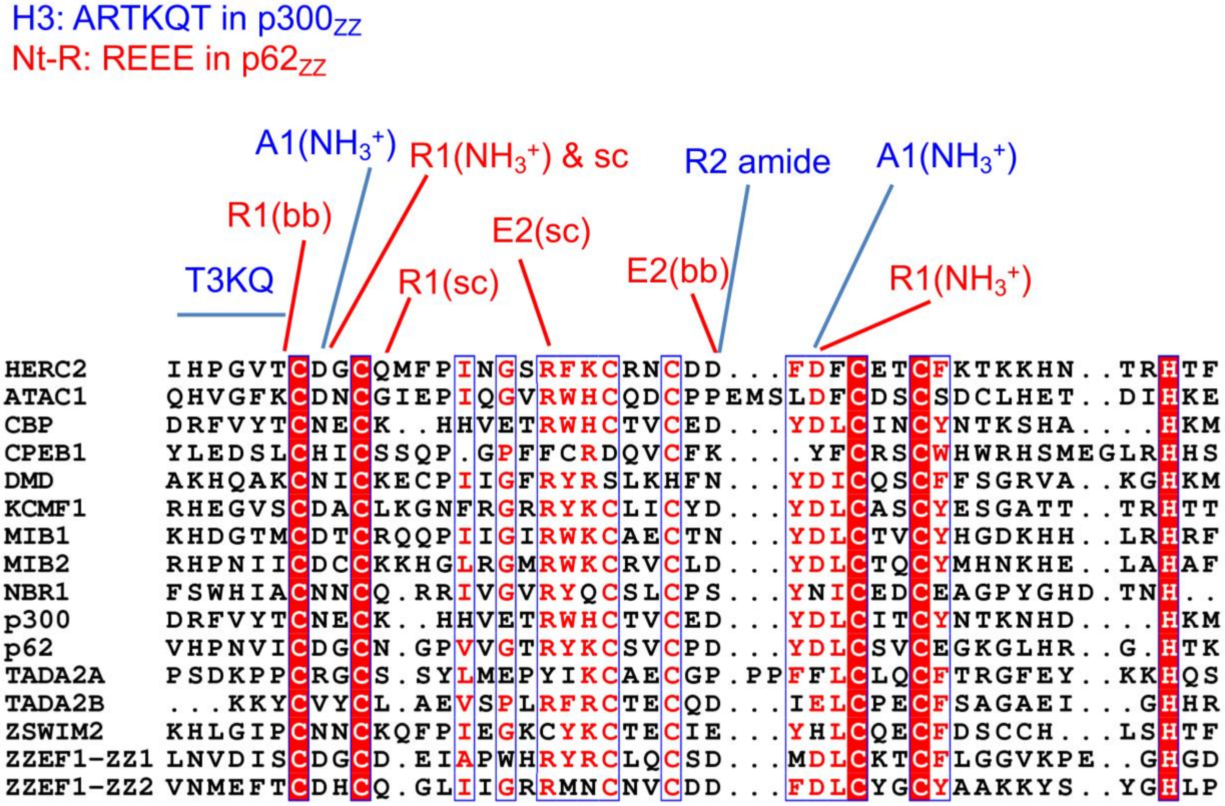

Supplementary Figure 7. Sequence alignment of the sixteen human ZZ domains.
